# Superior 5-Year Survival in Heart Transplant Recipients with Pre-Transplant Dilated Cardiomyopathy Versus Ischemic Cardiomyopathy

**DOI:** 10.3390/diagnostics16183032

**Published:** 2026-09-18

**Authors:** Alex Haag, Rasmus Rivinius, Matthias Helmschrott, Ann-Kathrin Rahm, Philipp Ehlermann, Norbert Frey, Fabrice F. Darche

**Affiliations:** Department of Cardiology, Angiology and Pneumology, Heidelberg University Hospital, 69120 Heidelberg, Germany

**Keywords:** coronary artery disease, familial dilated cardiomyopathy, idiopathic dilated cardiomyopathy, immunosuppression, mortality

## Abstract

**Background/Objectives**: Because donor-heart availability remains limited, adequate selection of candidates for heart transplantation (HTX) is essential for post-transplant success. Differences in the underlying etiology leading to HTX may play a relevant prognostic role. We compared the post-transplant outcomes of HTX recipients with pre-transplant dilated cardiomyopathy (DCM) and ischemic cardiomyopathy (ICM). **Methods**: We performed a retrospective single-center observational study of adult patients who underwent HTX at Heidelberg Heart Center between 1998 and 2020 excluding patients with repeat HTX. Patients were stratified according to pre-transplant DCM or ICM etiology. Our analysis comprised donor and recipient data, immunosuppressive therapy, post-transplant concomitant medications, and 5-year post-transplant mortality including causes of death. **Results**: This study included 158 patients with pre-transplant ICM (40.8%) and 229 patients with pre-transplant DCM (59.2%). HTX recipients with pre-transplant DCM had a significantly lower post-transplant 5-year mortality (27.5% versus 41.1%, *p* = 0.005). Multivariate analysis including 15 variables indicated a reduced risk of 5-year post-transplant mortality in individuals with pre-transplant DCM (HR: 0.478, 95% CI: 0.235–0.972, *p* = 0.042). A subgroup analysis of 62 HTX recipients with pre-transplant familial DCM and 167 HTX recipients with pre-transplant idiopathic DCM showed a significantly lower 5-year post-transplant mortality in HTX recipients with pre-transplant familial DCM (14.5% versus 32.3%, *p* = 0.007). A second multivariate analysis showed a reduced risk of 5-year post-transplant mortality in HTX recipients with pre-transplant familial DCM (HR: 0.380, 95% CI: 0.179–0.807, *p* = 0.012). **Conclusions**: HTX recipients with pre-transplant DCM, particularly familial DCM, showed superior 5-year post-transplant survival.

## 1. Introduction

Heart failure (HF) is a clinical syndrome defined by the inability of the myocardium to maintain a cardiac output sufficient to meet the metabolic demands of peripheral tissues, manifesting as distinct clinical signs and symptoms [1,2,3]. Despite significant advances in contemporary HF management, including guideline-directed pharmacological therapy, mechanical circulatory support, and catheter-based or surgical interventions, HF remains a leading cause of cardiovascular morbidity and mortality globally [1,2,3,4,5]. For patients with end-stage HF who remain symptomatic despite maximal treatment, heart transplantation (HTX) continues to be the gold standard and often the only life-saving treatment option [6,7]. However, a major challenge in contemporary transplant medicine is the limited availability of donor organs [7,8]. In addition, post-transplant outcomes can be influenced by recipient characteristics, underlying cardiac pathology, and post-transplant complications [2].

The two principal etiologies among HTX recipients are ischemic cardiomyopathy (ICM), resulting primarily from coronary artery disease (CAD), and dilated cardiomyopathy (DCM), which can be classified as familial/genetic or idiopathic/acquired [1,2,3,9,10,11,12,13,14]. These etiological subtypes differ substantially in pathophysiological mechanisms, demographic characteristics, and associated comorbidities: ICM is characterized by a higher burden of advanced atherosclerosis and cardiovascular risk factors, whereas DCM more frequently affects younger individuals and is generally associated with fewer systemic comorbidities [1,2,3,9,10,11,12,13,14,15,16,17,18,19].

The underlying etiology of cardiomyopathy may influence post-transplant outcomes, implying that ICM and DCM as indications for HTX might carry different prognostic weight. However, the influence of HF etiology on post-transplant outcomes remains controversial [20,21,22,23].

Given the limited and sometimes conflicting evidence regarding the impact of HF etiology on outcomes after HTX, this study aimed to investigate potential differences in post-transplant outcomes between HTX recipients with pre-transplant ICM and DCM, with particular emphasis on familial and idiopathic DCM subtypes.

## 2. Patients and Methods

### 2.1. Patients

All procedures undertaken within the scope of this study adhered to the ethical principles outlined in the Declaration of Helsinki. Ethics approval was granted by the Institutional Review Board of Heidelberg University (Heidelberg, Germany; reference S-286/2015, version 1.2; approval granted on 28 July 2020). Written informed consent was obtained from HTX recipients for the use of their clinical data for clinical and scientific purposes within the Heidelberg HTX Registry. Due to the retrospective, single-center study design and the reliance on routinely acquired clinical data, no further informed consent was required for the present study in accordance with the approved protocol [24,25,26,27].

We conducted a retrospective review of medical records from adult patients (≥18 years) who underwent HTX at the Heidelberg Heart Center (Germany) between 1998 and 2020. First, we stratified patients by the underlying etiology leading to HTX. Only patients with a pre-transplant diagnosis of ICM or DCM were included in the study cohort. Patients with pre-transplant hypertrophic cardiomyopathy, restrictive cardiomyopathy, arrhythmogenic right ventricular cardiomyopathy, or valvular cardiomyopathy were excluded. Patients who underwent repeat HTX were also excluded from this study. Second, HTX recipients with a prior diagnosis of DCM were further stratified into those with familial DCM and those with idiopathic DCM. Familial DCM was defined according to the European Society of Cardiology (ESC) Guidelines for the management of cardiomyopathies [28]. Specifically, familial DCM was diagnosed if (i) DCM was present in one or more first- or second-degree relatives, or (ii) an otherwise unexplained sudden cardiac death had occurred in a first-degree relative with a post-mortem or previously established diagnosis of DCM [28]. The family history was obtained from the routine pre-transplant evaluation and corresponding documents from the medical records; results of genetic testing were considered when available. HTX recipients with DCM who did not fulfill these criteria were classified as having idiopathic DCM.

### 2.2. Follow-Up

Post-transplant surveillance of HTX recipients was carried out following the standardized management protocol implemented at the Heidelberg Heart Center [24,25,26,27]. After discharge from the index hospitalization, HTX recipients were enrolled in a structured outpatient follow-up program consisting of monthly assessments during the initial six-month period after HTX. This was followed by a reduced visit frequency of every two months through the remainder of the first post-transplant year. In subsequent years, routine evaluations were conducted three to four times per year, supplemented by additional consultations when warranted by the patient’s clinical condition [24,25,26,27].

Follow-up visits were systematically conducted using a multimodal approach that encompassed clinical assessment, including medical history and physical examination, as well as laboratory investigations with monitoring of immunosuppressive drug levels. Cardiac evaluation consisted of resting 12-lead electrocardiography, transthoracic echocardiography, and routinely scheduled endomyocardial biopsy. Furthermore, annual surveillance included chest radiography and 24 h ambulatory ECG monitoring. Surveillance for CAD of the cardiac allograft comprised routine coronary angiography within 1, 2, and 5 years after HTX, irrespective of the underlying etiology, with additional angiography whenever clinically indicated. CAD was defined as a luminal diameter stenosis ≥50% in at least one coronary artery. Coronary stenting was performed in the presence of a severe stenosis (typically ≥70% luminal narrowing) of one or more major coronary arteries or of objective evidence of myocardial ischemia on non-invasive stress testing. Complete longitudinal data were obtained for all HTX recipients [24,25,26,27].

### 2.3. Post-Transplant Pharmacotherapy

Immunosuppressive and adjunct pharmacotherapy after HTX was implemented in accordance with the institutional protocol of the Heidelberg Heart Center [24,25,26,27]. Induction treatment generally consisted of anti-thymocyte globulin–based regimens. Maintenance immunosuppression evolved over the study period: initially, patients received cyclosporine A in combination with azathioprine; from 2001, azathioprine was replaced by mycophenolic acid; and from 2006 onward, tacrolimus was progressively introduced in place of cyclosporine A. Corticosteroids were administered to all recipients, with subsequent tapering and discontinuation targeted at approximately six months post-transplant, provided the clinical course allowed [24,25,26,27].

### 2.4. Statistical Analysis

Statistical analysis was carried out using MedCalc (Version 23.2.1; MedCalc Software Ltd., Ostend, Belgium). Selection of statistical tests (Student’s *t*-test, chi-square test, Mann–Whitney U test, or Fisher’s exact test) was guided by the type of data and the specific analytical objective. Continuous variables were reported as means accompanied by standard deviations (SD). Categorical variables were expressed as absolute counts and relative proportions. Effect estimates were reported as differences between groups (mean differences for continuous variables and differences in proportions for categorical variables) with corresponding 95% confidence intervals (CI). Survival following HTX was evaluated using Kaplan–Meier survival analysis, with group comparisons performed via the log-rank test. Figures and graphical representations were created with CorelDRAW Graphics Suite 2025 (Version 26.2.0.170; Corel Corporation, Ottawa, ON, Canada). Statistical significance was defined as a *p*-value < 0.050 [24,25,26,27].

Detailed univariate analyses were conducted to evaluate differences between HTX recipients with a prior diagnosis of ICM and those with DCM, as well as to compare HTX recipients with pre-transplant familial DCM to those with idiopathic DCM. The variables included in these comprehensive analyses comprised recipient-related characteristics, prior history of cardiac surgery, donor-specific parameters, donor–recipient sex mismatch, perioperative parameters, immunosuppressive regimens, and concomitant pharmacological treatments assessed during the index hospital stay following HTX [24,25,26,27].

The primary endpoint of this study was all-cause mortality within five years after HTX. Comparative analyses were performed between HTX recipients with a prior diagnosis of ICM and those with DCM, as well as between HTX recipients with familial DCM and those with idiopathic DCM. Causes of death occurring within the 5-year follow-up period were categorized as graft failure, acute rejection, infection/sepsis, malignancy, and thromboembolic event/bleeding [24,25,26,27].

In order to assess the impact of DCM as the underlying etiology leading to HTX on 5-year post-transplant mortality, a multivariate Cox proportional hazards regression model was constructed using all 387 HTX recipients. This model included all variables that demonstrated statistical significance in univariate analyses between HTX recipients with pre-transplant DCM and pre-transplant ICM. Variables were thus selected on the basis of the measured baseline imbalances between the compared groups rather than being defined a priori. In total, the following 15 variables were incorporated into the final analysis: recipient DCM before HTX, recipient age, recipient male sex, recipient body mass index (BMI), recipient arterial hypertension, recipient dyslipidemia, recipient diabetes mellitus, recipient chronic obstructive pulmonary disease (COPD), recipient history of smoking, recipient overall open-heart surgery, recipient coronary artery bypass graft (CABG) surgery, recipient ventricular assist device (VAD) surgery, donor age, donor (m) to recipient (f) transplant mismatch, and recipient beta-blocker therapy after HTX. Although prior CABG is intrinsically linked to ICM and none of the HTX recipients with DCM before HTX had undergone CABG, we used prior CABG in this model for two reasons. First, it identifies patients with ICM before HTX with extensive multivessel CAD and systemic atherosclerosis, and omitting it would disregard relevant heterogeneity in disease severity within the ICM group. Second, prior CABG entails the additional risk of a redo sternotomy, including pericardial adhesions and altered anatomical planes. In addition, a second multivariate Cox proportional hazards regression model was performed to specifically evaluate the impact of familial DCM as the underlying etiology leading to HTX on 5-year post-transplant mortality, including the 229 HTX recipients with pre-transplant DCM. This model included all variables that demonstrated statistical significance in univariate analyses between HTX recipients with pre-transplant familial DCM and pre-transplant idiopathic DCM. Altogether, the following 8 variables were incorporated into the final analysis: recipient familial DCM before HTX, recipient age, donor age, donor male sex, ischemic time, recipient azathioprine therapy after HTX, recipient beta-blocker therapy after HTX, and recipient calcium channel blocker therapy after HTX. The proportional hazards assumption was evaluated for all variables included in both multivariate Cox proportional hazards regression models, and no relevant violation was observed. The variable ‘recipient mycophenolic acid therapy after HTX’ was omitted because it is the complement of the variable ‘recipient azathioprine therapy after HTX’ within this cohort. Moreover, post-transplant medications can serve as either mediators or markers of different treatment eras. For instance, post-HTX beta-blocker and calcium-channel blocker therapies may highlight distinct clinical trajectories and complication profiles between the ICM and DCM cohorts. Conversely, the historical shift from azathioprine to mycophenolic acid may reflect the broader evolution toward modern immunosuppressive regimens. Finally, to account for potential temporal (era) effects over the prolonged study period, a sensitivity analysis was performed in the subgroup of HTX recipients who received a maintenance immunosuppressive regimen consisting of tacrolimus and mycophenolic acid. This subgroup reflects the standardized therapeutic approach adopted from 2006 onward following the modification of institutional immunosuppressive protocols [24,25,26,27]. To further address potential temporal effects, we divided the HTX cohort into three eras of comparable sample size (1998–2005, 2006–2011, and 2012–2020) and analyzed the distribution of pre-transplant ICM and DCM among HTX recipients.

## 3. Results

### 3.1. Demographics

Following the application of the predefined exclusion criteria, 387 patients who underwent HTX were eligible for inclusion in the study. Among the included HTX recipients, 158 individuals (40.8%) had been previously diagnosed with ICM, while 229 individuals (59.2%) had a history of DCM. Among HTX recipients with a pre-transplant diagnosis of DCM, 62 patients (27.1%) were classified as having familial DCM, whereas 167 patients (72.9%) were classified as having idiopathic DCM prior to HTX.

Patients with a pre-transplant diagnosis of ICM had a higher recipient age (56.4 ± 6.8 years versus 50.0 ± 11.6 years, *p* < 0.001), a higher proportion of male recipients (87.3% versus 73.8%, *p* = 0.001), a higher recipient BMI (26.0 ± 4.1 kg/m^2^ versus 25.1 ± 4.1 kg/m^2^, *p* = 0.038), a higher percentage of recipient arterial hypertension (96.2% versus 33.6%, *p* < 0.001), a higher percentage of recipient dyslipidemia (98.1% versus 52.0%, *p* < 0.001), a higher percentage of recipient diabetes mellitus (44.9% versus 28.8%, *p* = 0.001), a higher percentage of recipient COPD (38.6% versus 20.5%, *p* < 0.001), a higher percentage of recipient history of smoking (81.0% versus 50.7%, *p* < 0.001), a higher percentage of recipient overall open-heart surgery before HTX (50.6% versus 28.8%, *p* < 0.001), a higher percentage of recipient CABG surgery before HTX (39.9% versus 0.0%, *p* < 0.001), and a higher donor age (45.5 ± 12.6 years versus 41.9 ± 13.7 years, *p* = 0.009). In contrast, HTX recipients with pre-transplant DCM showed a higher percentage of recipient VAD surgery before HTX (21.0% versus 11.4%, *p* = 0.014) and male-donor-to-female-recipient sex mismatch (5.7% versus 1.3%, *p* = 0.027). Demographics stratified by DCM versus ICM before HTX are shown in Table 1.

Subgroup analysis of HTX recipients with a history of DCM showed that individuals with familial DCM had a lower recipient age (46.1 ± 11.1 years versus 51.5 ± 11.4 years, *p* = 0.001), a higher donor age (45.8 ± 10.7 years versus 40.5 ± 14.4 years, *p* = 0.003), a lower percentage of donor male sex (21.0% versus 45.5%, *p* = 0.001), and a longer ischemic time (255.9 ± 60.4 min versus 234.6 ± 60.7 min, *p* = 0.020). Demographics stratified by familial DCM versus idiopathic DCM before HTX are given in Table 2.

### 3.2. Initial Post-Transplant Medications

Comparative analysis of the initial post-transplant pharmacotherapy between HTX recipients with pre-transplant ICM and DCM demonstrated no statistically significant differences, with the exception of recipient beta-blocker therapy following HTX, which was more frequently administered in patients with pre-transplant ICM than in those with DCM (24.7% versus 16.2%, *p* = 0.038). Initial post-transplant medication regimens stratified according to pre-transplant DCM versus ICM are presented in Table 3.

Subgroup analysis comparing patients with pre-transplant familial DCM and idiopathic DCM prior to HTX revealed no statistically significant differences in the use of cyclosporine A or tacrolimus as immunosuppressive drug therapy, but individuals with pre-transplant familial DCM more frequently received mycophenolic acid following HTX (91.9% versus 73.7%, *p* = 0.003), while individuals with pre-transplant idiopathic DCM more often received azathioprine after HTX (26.3% versus 8.1%, *p* = 0.003). In addition, patients with pre-transplant familial DCM showed a higher percentage of beta-blocker therapy following HTX (27.4% versus 12.0%, *p* = 0.005), whereas individuals with pre-transplant idiopathic DCM had a higher percentage of calcium channel blocker therapy following HTX (34.1% versus 16.1%, *p* = 0.008). Initial post-transplant medication regimens stratified by familial versus idiopathic DCM prior to HTX are shown in Table 4.

### 3.3. Post-Transplant Mortality and Causes of Death

**Figure 1:**
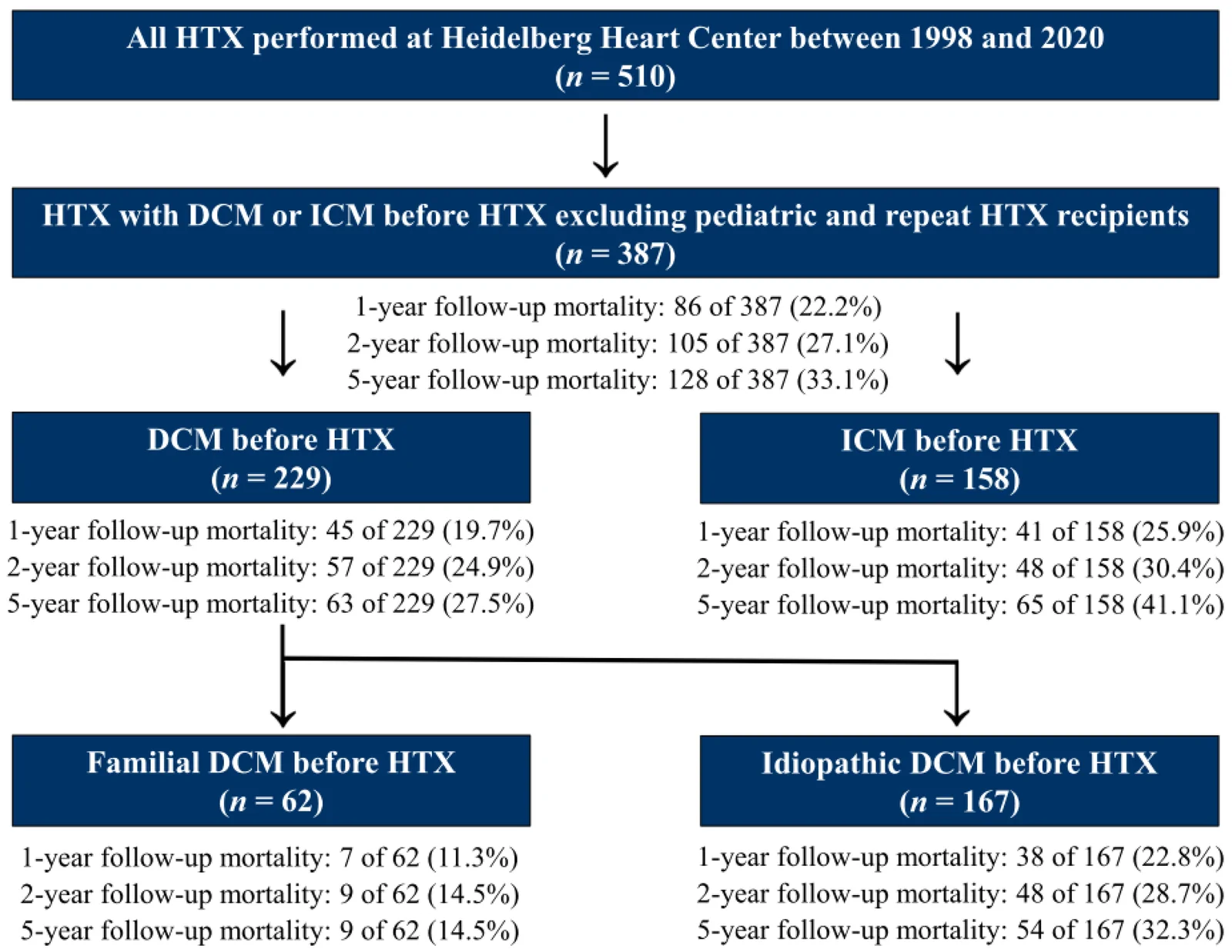
Overview of post-transplant mortality stratified by DCM/ICM before HTX and familial/idiopathic DCM before HTX. HTX recipients with familial DCM prior to HTX had the lowest 1-, 2-, and 5-year post-transplant mortality, followed by HTX recipients with overall DCM prior to HTX, and HTX recipients with idiopathic DCM prior to HTX. HTX recipients with ICM prior to HTX showed the highest 1-, 2-, and 5-year mortality after HTX. DCM = dilated cardiomyopathy; HTX = heart transplantation; ICM = ischemic cardiomyopathy; *n* = number.

Regarding the primary endpoint of the study, individuals with pre-transplant familial DCM exhibited the lowest post-transplant mortality rates at 1 year (11.3%), 2 years (14.5%), and 5 years (14.5%). These were followed by individuals with overall pre-transplant DCM (1-year: 19.7%, 2-year: 24.9%, 5-year: 27.5%) and those with pre-transplant idiopathic DCM (1-year: 22.8%, 2-year: 28.7%, 5-year: 32.3%). In contrast, recipients with pre-transplant ICM demonstrated the highest mortality rates following HTX at 1 year (25.9%), 2 years (30.4%), and 5 years (41.1%). An overview of post-transplant mortality stratified by pre-transplant DCM versus ICM and familial DCM versus idiopathic DCM is presented in Figure 1.

Analysis of 5-year cause-specific mortality after HTX revealed a significantly higher proportion of infection- or sepsis-related mortality among individuals with pre-transplant ICM compared with those with pre-transplant DCM (22.2% versus 14.0%, *p* = 0.037). No statistically significant differences in 5-year post-transplant mortality were observed between the groups with respect to graft failure, acute rejection, malignancy, and thromboembolic event/bleeding (all *p* ≥ 0.050). Subgroup analysis comparing patients with pre-transplant familial DCM and idiopathic DCM revealed no statistically significant differences in 5-year post-transplant mortality with respect to graft failure, acute rejection, infection/sepsis, malignancy, and thromboembolic event/bleeding (all *p* ≥ 0.050). 5-year cause-specific mortality after HTX is summarized in Table 5 and Table 6.

**Figure 2:**
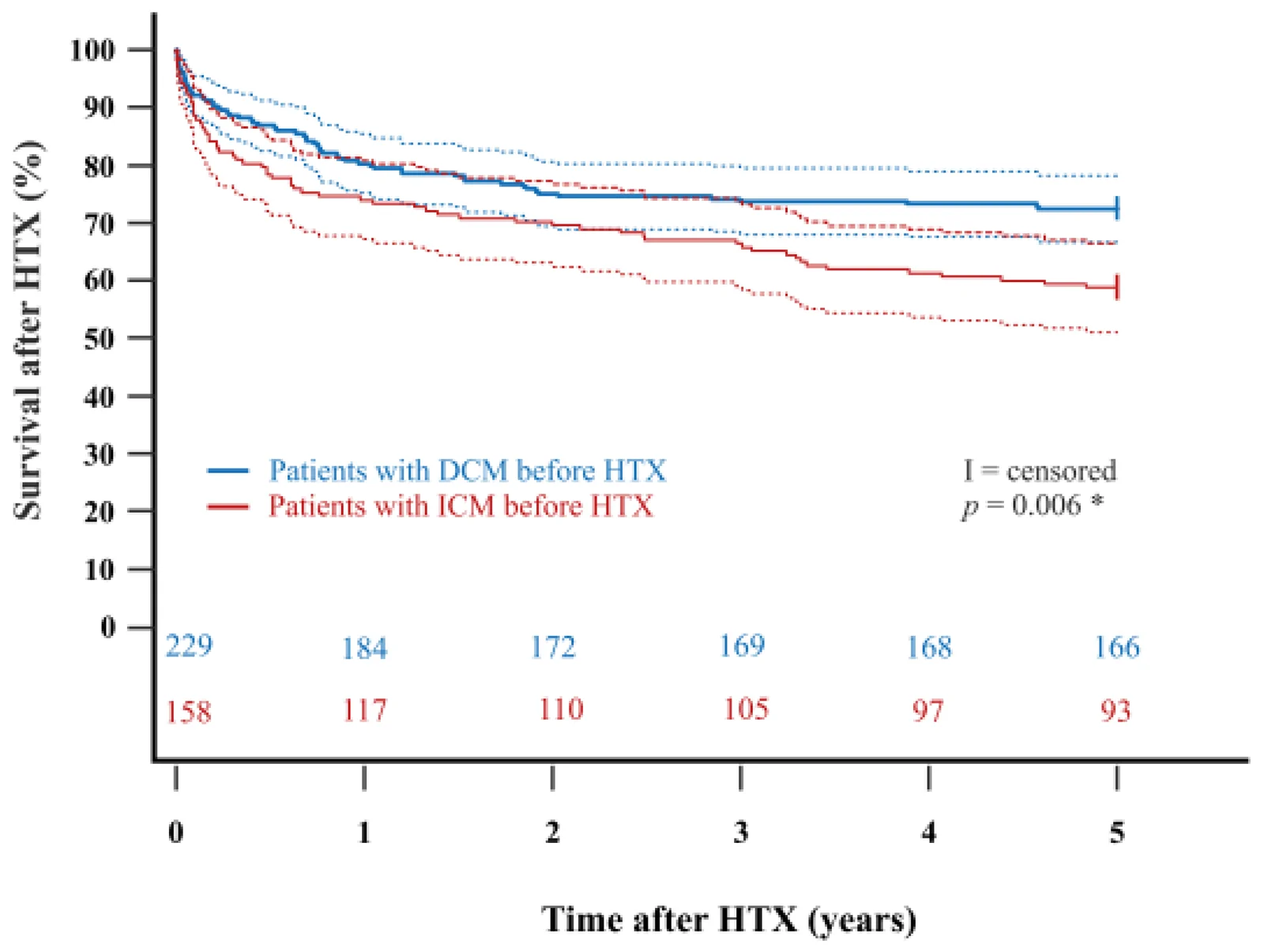
5-year post-transplant survival between patients with DCM before HTX and patients with ICM before HTX (Kaplan–Meier estimator). HTX recipients with DCM prior to HTX showed a significantly better 5-year post-transplant survival than HTX recipients with ICM prior to HTX (log-rank test *p* = 0.006). Dashed lines represent the 95% confidence interval around the respective survival curve. DCM = dilated cardiomyopathy; HTX = heart transplantation; ICM = ischemic cardiomyopathy; * = statistically significant (*p* < 0.050).

**Figure 3:**
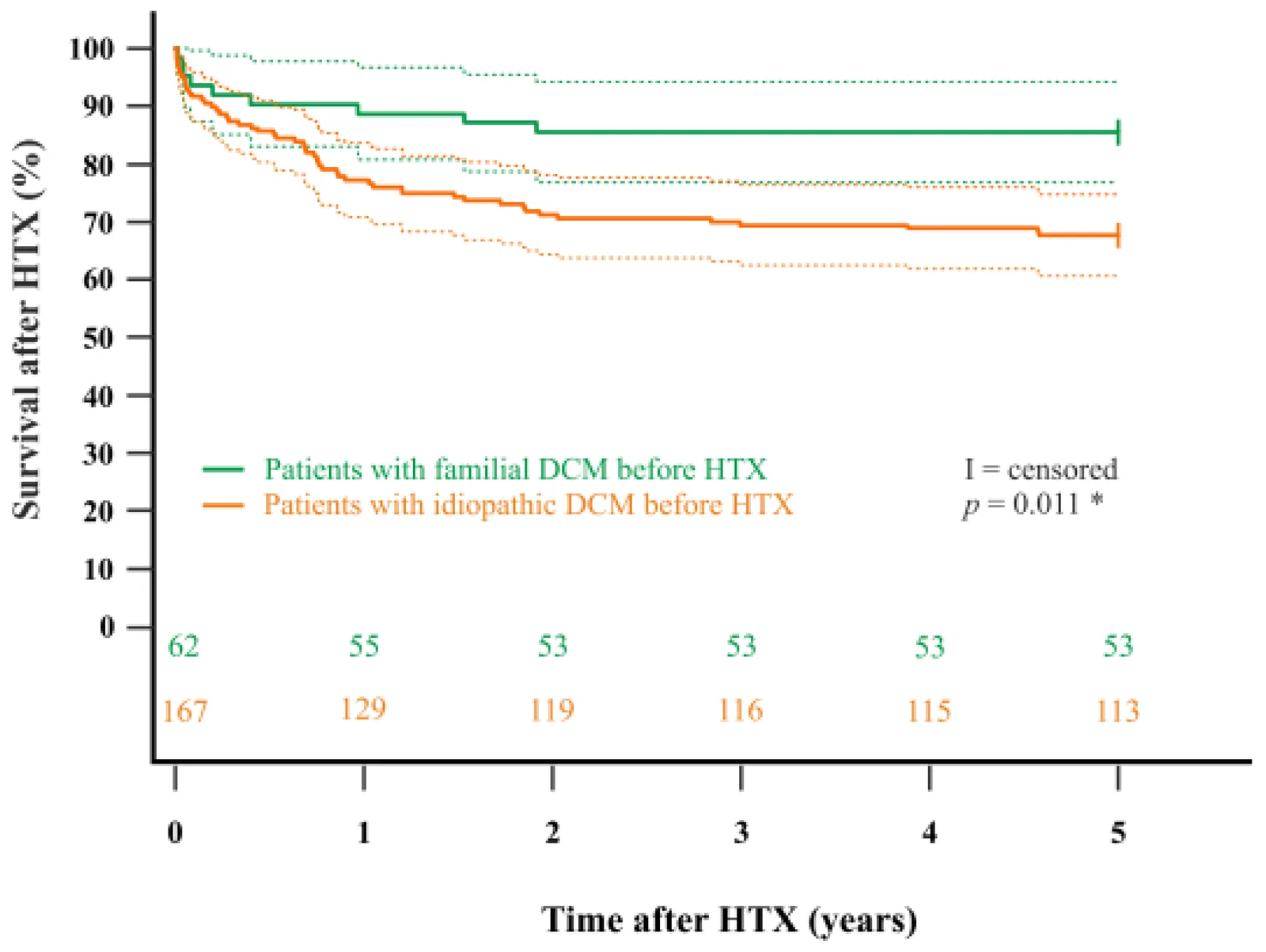
5-year post-transplant survival between patients with familial DCM before HTX and patients with idiopathic DCM before HTX (Kaplan–Meier estimator). HTX recipients with familial DCM prior to HTX showed a significantly better 5-year post-transplant survival than HTX recipients with idiopathic DCM prior to HTX (log-rank test *p* = 0.011). Dashed lines represent the 95% confidence interval around the respective survival curve. DCM = dilated cardiomyopathy; HTX = heart transplantation; * = statistically significant (*p* < 0.050).

The Kaplan–Meier estimator demonstrated a significantly superior 5-year post-transplant survival among individuals with pre-transplant DCM compared with those with pre-transplant ICM (log-rank test *p* = 0.006). Subgroup analysis of HTX recipients with pre-transplant DCM revealed a significantly better 5-year post-transplant survival in individuals with familial DCM prior to HTX compared with those with idiopathic DCM prior to HTX (log-rank test *p* = 0.011). Kaplan–Meier survival curves are shown in Figure 2 and Figure 3.

### 3.4. Multivariate Analysis

Multivariate analysis for post-transplant mortality, including 15 variables, showed that HTX recipients with pre-transplant DCM had a significantly lower risk of death within five years after HTX (hazard ratio (HR): 0.478, 95% CI: 0.235–0.972, *p* = 0.042). In contrast, recipient age (HR: 1.031, 95% CI: 1.007–1.055, *p* = 0.011), recipient COPD (HR: 4.202, 95% CI: 2.736–6.452, *p* < 0.001), and recipient overall open-heart surgery before HTX (HR: 1.998, 95% CI: 1.098–3.636, *p* = 0.023) were identified as independent predictors of increased 5-year post-transplant mortality. The remaining variables were not significantly associated with 5-year mortality after HTX in the multivariate analysis. Detailed results of the multivariate analysis evaluating 5-year mortality after HTX with a focus on pre-transplant DCM are presented in Table 7.

Furthermore, a multivariate analysis of post-transplant mortality was performed with a specific focus on HTX recipients with familial DCM prior to HTX. Pre-transplant familial DCM was associated with a significantly lower risk of death within five years after HTX (HR: 0.380, 95% CI: 0.179–0.807, *p* = 0.012). Additionally, male donor sex was associated with reduced 5-year post-transplant mortality (HR: 0.552, 95% CI: 0.317–0.961, *p* = 0.036), while longer ischemic time (HR: 1.006, 95% CI: 1.001–1.011, *p* = 0.017) and post-transplant azathioprine therapy (HR: 2.541, 95% CI: 1.192–5.418, *p* = 0.016) were identified as independent predictors of increased 5-year post-transplant mortality. No significant associations with 5-year post-transplant mortality were observed for the remaining variables included in the multivariate analysis. Comprehensive results of the multivariate analysis assessing 5-year mortality after HTX with a specific focus on pre-transplant familial DCM are summarized in Table 8.

### 3.5. Sensitivity Analysis and Analysis of Transplant Eras

Given the prolonged study period, a sensitivity analysis was conducted to assess the potential impact of an era effect in the subgroup of HTX recipients treated with tacrolimus and mycophenolic acid–based immunosuppression (238 of 387 HTX recipients [61.5%]). This analysis showed a significantly lower 5-year post-transplant mortality among individuals with pre-transplant DCM compared to those with pre-transplant ICM (27.1% versus 41.8%, *p* = 0.018). In addition, individuals with familial DCM before HTX had a significantly lower 5-year post-transplant mortality than individuals with idiopathic DCM before HTX (15.9% versus 32.3%, *p* = 0.043). Therefore, the results of this subgroup analysis were consistent with those of the overall cohort, indicating the stability of the observed associations and suggesting that the findings were unlikely to be substantially influenced by temporal changes in clinical practice.

In addition, to account for potential temporal effects, the HTX cohort was divided into three similarly sized transplant eras (1998–2005, 2006–2011, and 2012–2020) to analyze the distribution of HTX recipients with pre-transplant ICM and DCM. There was no statistically significant difference between HTX recipients with pre-transplant DCM or ICM in the era of 1998–2005 (32.7% versus 33.5%, *p* = 0.871), in the era of 2006–2011 (34.1% versus 40.5%, *p* = 0.196), and in the era of 2012–2020 (33.2% versus 26.0%, *p* = 0.128).

### 3.6. CAD and Coronary Stenting After HTX

Angiographically detected CAD (stenosis ≥ 50%) was present in 17 of 387 HTX recipients (4.4%) within the first year after HTX and increased to 48 HTX recipients (12.4%) within 2 years after HTX and 75 HTX recipients (19.4%) within 5 years after HTX. Within both groups, the proportion of CAD increased significantly by 5 years after HTX (DCM: *p* < 0.001; ICM: *p* < 0.001). The rate of coronary stenting also increased significantly within both groups by 5 years after HTX (DCM: *p* < 0.001; ICM: *p* = 0.002). There were no statistically significant differences between HTX recipients with pre-transplant DCM and ICM regarding the proportion of CAD within the first year after HTX (5.2% versus 3.2%, *p* = 0.327), within 2 years after HTX (12.2% versus 12.7%, *p* = 0.899), or within 5 years after HTX (19.2% versus 19.6%, *p* = 0.921). Likewise, coronary stenting was performed at similar rates in both groups within the first year after HTX (1.3% versus 0.6%, *p* = 0.517), within 2 years after HTX (6.1% versus 2.5%, *p* = 0.100), and within 5 years after HTX (8.3% versus 7.6%, *p* = 0.803). Results of cardiac catheterization stratified by DCM versus ICM before HTX are presented in Table 9.

In the subgroup analysis of HTX recipients with pre-transplant DCM, no statistically significant differences were observed between familial and idiopathic DCM regarding the proportion of CAD within the first year after HTX (9.7% versus 3.6%, *p* = 0.066), within 2 years after HTX (12.9% versus 12.0%, *p* = 0.849), or within 5 years after HTX (19.4% versus 19.2%, *p* = 0.974), or regarding coronary stenting within the first year after HTX (3.2% versus 0.6%, *p* = 0.120), within 2 years after HTX (8.1% versus 5.4%, *p* = 0.453), or within 5 years after HTX (11.3% versus 7.2%, *p* = 0.317). While the proportions of CAD and coronary stenting increased significantly over time in HTX recipients with idiopathic DCM before HTX, the corresponding increases did not reach statistical significance in the smaller subgroup with familial DCM before HTX. Results of cardiac catheterization stratified by familial versus idiopathic DCM before HTX are given in Table 10.

## 4. Discussion

### 4.1. Post-Transplant Survival of HTX Recipients with Pre-Transplant DCM and ICM

To our knowledge, only limited data exist regarding post-transplant mortality differences between patients transplanted for DCM and ICM. In our cohort, HTX recipients with pre-transplant DCM demonstrated a significantly lower 5-year mortality following HTX compared with HTX recipients with pre-transplant ICM (27.5% versus 41.1%, *p* = 0.005). Furthermore, patients with pre-transplant familial DCM demonstrated lower post-transplant 5-year mortality compared with HTX recipients diagnosed with idiopathic DCM (14.5% versus 32.3%, *p* = 0.007).

Although research data on the comparison of HTX recipient outcomes between pre-transplant ICM versus pre-transplant DCM remain limited, one of the largest available datasets is the International Society for Heart and Lung Transplantation (ISHLT) registry’s annual report [29]. The 2025 annual report did not identify a statistically significant association between the diagnosis of pre-transplant ICM and higher 1-year mortality after HTX compared with the diagnosis of pre-transplant non-ICM (HR: 1.06, 95% CI: 0.98–1.14, *p* = 0.15). However, this report compared neither HTX recipients with pre-transplant ICM versus DCM, nor outcomes based on pre-transplant familial DCM versus idiopathic DCM etiology [29].

Similarly, a study by Gungor and colleagues [21] compared post-transplant outcomes of 38 HTX recipients with ICM and 86 HTX recipients with idiopathic DCM, who received HTX between 1998 and 2011, without examining familial DCM separately. They reported no statistically significant differences in 5-year post-transplant survival between the groups, although HTX recipients with pre-transplant idiopathic DCM showed a higher 5-year post-transplant survival than HTX recipients with pre-transplant ICM (62.3% versus 54.2%, *p* = 0.57). The absence of statistical significance may be a result of the rather small sample size within their study cohort [21].

In addition, Oehler and colleagues [20] compared post-transplant outcomes of 218 HTX recipients, 92 HTX recipients with ICM and 126 HTX recipients with non-ICM, who underwent HTX between 2010 and 2021. Their findings did not identify a statistically significant association between the underlying etiology and 1-year survival after HTX (76.1% versus 76.5%, *p* = 0.59). This contrasts with our findings and may reflect differences in cohort composition, transplant era, sample size, or follow-up care [20].

In line with our findings, Martinelli and colleagues [23] observed a superior 5-year post-transplant survival in 147 HTX recipients with pre-transplant DCM compared with 128 HTX recipients with pre-transplant ICM (87.0% versus 70.8%, *p* < 0.01). Furthermore, Antunes and colleagues [22] found a significantly better left ventricular ejection fraction (LVEF) at 1-year follow-up after HTX in HTX recipients with pre-transplant idiopathic DCM compared with HTX recipients with pre-transplant ICM. The authors suggested a strong correlation between pre-transplant etiology and the systolic function of the cardiac allograft at 1-year follow-up [22].

These findings suggest that the underlying etiology of end-stage HF may remain relevant for long-term prognosis even after a successful HTX. However, the more favorable cardiovascular risk profile of HTX recipients with pre-transplant DCM, particularly familial DCM, may inherently contribute to their superior post-transplant survival outcomes—an association that warrants further investigation.

### 4.2. Cardiovascular Risk Profiles of HTX Recipients with Pre-Transplant DCM and ICM

Recipients transplanted for ICM exhibited a substantially different baseline cardiovascular risk profile compared with recipients transplanted for DCM. In our cohort, HTX recipients presenting with pre-transplant ICM were older and displayed a higher prevalence of cardiovascular risk factors, including an elevated average BMI, dyslipidemia, previous cardiac surgeries, and comorbid COPD. These findings are consistent with data published by Gungor and colleagues [21], who evaluated 38 HTX recipients with pre-transplant ICM against 86 HTX recipients with idiopathic DCM. Consistently, their findings demonstrated significantly higher age, increased BMI, and a higher rate of hyperlipidemia among HTX recipients in the pre-transplant ICM cohort [21]. Furthermore, careful selection of elderly candidates is essential, as pre-transplant frailty is linked to worse post-transplant survival [30].

Additionally, the higher rate of diabetes mellitus observed among HTX recipients with pre-transplant ICM represents a finding of particular clinical relevance [27]. Diabetes mellitus is characterized by accelerated atherosclerosis, endothelial dysfunction, chronic inflammation, and microvascular disease [31,32,33], and its systemic manifestations continue to adversely affect clinical outcomes after HTX. Moreover, our study cohort revealed that HTX recipients with pre-transplant ICM more frequently presented with concomitant COPD, a comorbidity inherently linked to an elevated cardiovascular burden and mortality [34,35]. In HTX recipients, the presence of COPD adversely affects clinical outcomes and is significantly associated with impaired post-transplant survival [26].

ICM is predominantly driven by atherosclerosis [36]. Due to the systemic nature of this pathology, polyvascular disease is highly prevalent and concomitant carotid and lower extremity peripheral artery disease in patients with ICM can significantly elevate the risk of subsequent adverse cardiovascular events [36]. Although HTX successfully addresses the localized myocardial failure and epicardial CAD, it does not mitigate the underlying systemic vascular pathology that drove initial disease development [24]. Consequently, HTX recipients with pre-transplant ICM may be predisposed to accelerated atherosclerosis of the graft’s epicardial vessels and remain highly vulnerable to extracardiac vascular complications, including ischemic stroke, peripheral arterial disease, and carotid artery stenosis [24]. In a comparative study including 46 HTX recipients with pre-transplant ICM and 119 HTX recipients with non-ischemic etiologies, Guddeti and colleagues [37] demonstrated via multivariate analysis that pre-transplant ICM was independently associated with both plaque progression (odds ratio (OR): 3.10; 95% CI: 1.17–9.36; *p* = 0.023) and long-term adverse events (HR: 2.02; 95% CI: 1.01–4.00; *p* = 0.048).

The unfavorable cardiovascular risk profile of HTX recipients with pre-transplant ICM may therefore represent an important contributor to the increased 5-year post-transplant mortality observed in our cohort. These findings suggest that post-transplant outcomes are influenced not only by the underlying HF etiology but also by the cumulative burden of systemic cardiovascular comorbidities.

### 4.3. The Potential Impact of Pre-Transplant DCM and ICM as Underlying Etiologies on the Selection Process for HTX Candidates

The significantly elevated 5-year post-transplant mortality observed among HTX recipients with pre-transplant ICM in the present cohort suggests that the underlying HF etiology may be an underrecognized determinant of long-term outcomes after HTX. Furthermore, stratified analysis revealed that familial DCM etiology was associated with superior 5-year post-transplant survival outcomes relative to idiopathic DCM. These findings suggest that HF etiology may provide additional prognostic information in pre-transplant risk stratification in the context of the critical scarcity of donor organs [7,8,38]. However, because etiology is closely intertwined with the broader clinical phenotype, including age, comorbidities, vascular disease burden, transplant urgency, and treatment era, it should not be considered in isolation but rather as one component of a comprehensive, multiparametric risk assessment during candidate selection.

While current HTX candidate selection integrates multidimensional recipient parameters—ranging from physiological functions to overall frailty [2]—the independent prognostic value of the underlying HF etiology remains highly debated [20,21,22,23].

DCM is defined as a myocardial pathology characterized by left ventricular dilatation with global or regional systolic dysfunction not explained solely by abnormal loading conditions or CAD [28,39]. In contemporary cohorts, pathogenic or likely pathogenic genetic variants are identified in up to 40% of patients diagnosed with DCM [28]. Consequently, first-degree relatives exhibit a significantly elevated predisposition to developing DCM [40]. In the absence of confirmatory genetic data within a kindred, a familial etiology of DCM is established if at least one of the following criteria is met: (i) the presence of DCM in one or more first- or second-degree relatives; or (ii) the occurrence of an otherwise unexplained sudden cardiac death at any age in a first-degree relative with a post-mortem or prior established diagnosis of DCM [28]. Crucially, the absence of an identified familial pathogenic variant or an abnormal pedigree does not preclude a genetic etiology of DCM, as de novo mutations may occur; consequently, genetic testing remains indicated if clinical suspicion persists [12]. Furthermore, accumulating evidence demonstrates that a subset of patients with familial DCM exhibits a phenotype characterized by rapid disease progression [41,42].

Given that DCM is a major etiology of HF and HTX globally, familial DCM constitutes a clinical entity of paramount importance. However, the precise prevalence of familial DCM among HF patients and HTX recipients remains elusive, as documented rates within the United Network for Organ Sharing (UNOS) registry are low, ranging from 2.7% to 3.1% [13,43]. This low prevalence of familial DCM among patients listed for HTX within the UNOS registry may be attributable to systemic underdiagnosis of the familial variant [13,43]. Extrapolating center-specific UNOS data from a large United States tertiary care academic medical center, Seidelmann and colleagues [43] demonstrated that comprehensive family history assessment and pedigree analysis identified a familial etiology in approximately 26% of patients with DCM.

In a UNOS registry analysis of 677 patients with familial DCM listed for HTX, Khayata and colleagues [13] observed that this cohort was characterized by a younger age at presentation and a lower male predominance compared with patients with ICM, which aligns with the findings of our study. Furthermore, the authors reported that post-transplant survival rates at 1, 3, and 5 years were significantly superior in HTX recipients with pre-transplant familial DCM (91%, 88%, and 80%, respectively) compared with those with pre-transplant ICM (89%, 82%, and 75%, respectively, *p* = 0.008). The authors postulated that this post-transplant survival advantage is mediated by a lower prevalence of hepatic and renal impairment within the familial DCM cohort. Conversely, the elevated mortality observed among patients with pre-transplant ICM is likely driven by a higher burden of concurrent cardiovascular and systemic comorbidities [13].

These observations align with a comparative analysis of the UNOS registry by Seidelmann and colleagues [43], which evaluated 492 patients with familial DCM and 15,599 patients with idiopathic DCM. The investigators demonstrated that the familial DCM cohort was characterized by a significantly younger recipient age (42.1 ± 13.1 years versus 48.5 ± 12.5 years, *p* = 0.0001), a lower percentage of male recipients (63.2% versus 68.9%, *p* = 0.007), and a lower percentage of recipient diabetes mellitus (7.8% versus 16.5%, *p* < 0.0001) [43].

In a comparative study of 16 HTX recipients with familial DCM and 168 HTX recipients with idiopathic DCM, Valantine and colleagues [41] observed a higher 5-year post-transplant survival rate in the familial cohort (85% versus 35%). However, the small number of patients with familial DCM (*n* = 16) limits the statistical power of this comparison [41].

Taken together, the favorable post-transplant outcomes observed in both our study cohort and previous studies [13,41,43] suggest that familial DCM recipients may constitute a subgroup particularly likely to benefit from HTX. As familial DCM is often associated with pathogenic genetic variants and may exhibit a more aggressive disease course prior to HTX [12,13,14,44,45,46], the identification of a genetic etiology may provide valuable prognostic information during HTX candidate evaluation. In addition, increasing evidence suggests that genotype-based approaches may provide valuable prognostic information beyond conventional phenotype-based classifications [47,48]. Furthermore, our findings suggest that the adverse prognostic impact associated with familial DCM before HTX may not persist once the native heart is replaced. One possible explanation is that HTX effectively removes the primary organ affected by the underlying genetic disease, thereby attenuating many of the disease-specific mechanisms responsible for progressive myocardial dysfunction. This may have important implications for evaluation and selection of HTX candidates. Therefore, given that genetic testing has become an integral part of routine clinical practice, recognition of familial DCM may contribute to improved risk stratification and help identify patients with a particularly favorable post-transplant prognosis.

### 4.4. CAD After HTX

Given the higher systemic atherosclerotic burden of HTX recipients with pre-transplant ICM, a higher incidence of CAD of the cardiac allograft might have been expected in this group [37]. In our cohort, however, the proportion of angiographically detected CAD increased substantially over time after HTX in both groups but did not differ between HTX recipients with pre-transplant DCM and ICM within 5 years after HTX (19.2% versus 19.6%, *p* = 0.921). Similarly, rates of coronary stenting were comparable between both groups, as well as between familial and idiopathic DCM. These findings suggest that the higher 5-year post-transplant mortality of HTX recipients with pre-transplant ICM is unlikely to be explained by a higher incidence of epicardial CAD of the cardiac allograft within the first five years after HTX. Instead, the excess mortality in this group may be more closely related to the extracardiac consequences of systemic atherosclerosis and associated comorbidities, such as COPD, diabetes mellitus, and older age, which is in line with the higher rate of infection/sepsis-related deaths in HTX recipients with pre-transplant ICM (22.2% versus 14.0%, *p* = 0.037). Nevertheless, two aspects should be considered when interpreting these data. First, coronary angiography is known to underestimate the extent of cardiac allograft vasculopathy compared with intravascular imaging, so that subclinical differences between the groups cannot be excluded. Second, because HTX recipients with pre-transplant ICM had a higher mortality during follow-up, fewer of these patients remained at risk for the development of CAD, which may have led to an underestimation of CAD in this group.

### 4.5. Study Limitations

The present findings are based on data obtained from the Heidelberg HTX Registry, representing a large single-center cohort of HTX recipients. Owing to the observational single-center design, certain inherent limitations could not be overcome, and the results should therefore be interpreted cautiously and in conjunction with the current body of evidence. However, the study benefits from the inclusion of detailed and systematically collected clinical data from 387 HTX recipients who underwent treatment and follow-up according to standardized institutional protocols. Moreover, no patients were lost to follow-up, ensuring comprehensive data sets. This standardized single-center approach helped to reduce practice heterogeneity, but selection bias and residual confounding remain possible [24,25,26,27].

In order to ensure adequate statistical power and the validity of the analyses, the study included patients who underwent HTX at the Heidelberg Heart Center between 1998 and 2020 with a minimum post-transplant follow-up of 5 years. Given the long recruitment period, changes in surgical strategies, perioperative management, and immunosuppressive treatment protocols over time may have influenced clinical outcomes after HTX, potentially resulting in era-related effects, such as the relatively low rates of statin (56.3%) and aspirin (16.3%) use in our cohort. Routine statin therapy for the prevention of cardiac allograft vasculopathy was less firmly established in the early years of our study period than it is today. Moreover, aspirin was not routinely administered to all HTX recipients but prescribed in the presence of a specific indication. To account for this potential source of heterogeneity, a sensitivity analysis was performed in the subgroup of HTX recipients treated with tacrolimus and mycophenolic acid, which has represented the institutional standard immunosuppressive regimen since 2006. Our sensitivity analysis of HTX recipients treated with tacrolimus and mycophenolic acid showed comparable findings, reinforcing the robustness and consistency of the overall study results. To address potential temporal confounding, HTX recipients with pre-transplant ICM and DCM were stratified into three similarly sized transplant eras (1998–2005, 2006–2011, and 2012–2020). Since no statistically significant differences were observed across these eras, transplant era was not included in the multivariate adjusted models, ensuring consistency with the remainder of our analyses.

Several methodological aspects of the multivariate analyses should be considered. First, the variables of the multivariate models were selected on the basis of statistically significant differences in univariate group comparisons rather than being defined a priori. This approach ensured that the measured baseline imbalances between the compared groups were accounted for, while variables that were comparably distributed between the groups had limited potential to confound the association between the underlying etiology and post-transplant mortality. However, this strategy may result in the omission of masked confounders and in overly optimistic estimates, and residual confounding by unmeasured variables cannot be excluded. Second, prior CABG surgery is intrinsically linked to ICM and was absent in HTX recipients with DCM; its effect estimate should therefore be interpreted as a marker of advanced coronary disease burden within the ICM group rather than as a general risk factor across both etiologies. Third, post-transplant medications were included as adjustment variables because they reflect the immunosuppressive treatment era and the clinical risk profile of the recipients; however, as they were recorded after HTX, they may also act as mediators on the pathway between etiology and mortality, and adjustment for them may have attenuated or distorted the estimated effect of etiology. The corresponding hazard ratio should therefore be interpreted with caution, as its precision may be overestimated. Nevertheless, the confidence intervals of this model did not indicate numerical instability, and the survival advantage of familial DCM was consistent across the Kaplan–Meier analysis, the multivariate analysis, and the sensitivity analysis restricted to HTX recipients treated with tacrolimus and mycophenolic acid.

Optimally, risk stratification would have included a more detailed classification of familial DCM into genetically confirmed and non-genetically confirmed cases of familial DCM among HTX recipients. In addition, truly familial cases with incomplete penetrance, variable expressivity, de novo variants, or an unknown family history may have been classified as idiopathic, whereas a positive family history due to shared non-genetic factors may have led to the classification of non-genetic cases as familial. However, owing to the retrospective nature of the study, the availability of such data was limited, as genetic information and related parameters were not routinely documented within the Heidelberg HTX Registry. Furthermore, the low rate of events (only 9 deaths occurred in the familial DCM before HTX group) bears the risk of overly optimistic estimates. However, to the best of our knowledge, this investigation constitutes one of the largest analyses to date—aside from the annual reports of the ISHLT and UNOS data—comparing post-transplant survival among HTX recipients with pre-transplant ICM and DCM as well as between HTX recipients with pre-transplant familial DCM and idiopathic DCM. Notably, HTX recipients with a prior diagnosis of DCM demonstrated a significantly superior 5-year post-transplant survival compared with those with pre-transplant ICM, with the most pronounced benefit observed in HTX recipients with a history of familial DCM. Although causal inferences cannot be drawn from this observational design, the magnitude and specificity of the observed associations, particularly in relation to differences in cardiovascular risk profiles, suggest a biologically plausible relationship that warrants further investigation. Confirmation of these findings will require large, prospective, multicenter studies, which should also evaluate whether targeted reduction in cardiovascular risk factors can improve long-term post-transplant survival.

## 5. Conclusions

In the context of the ongoing critical shortage of donor organs, rigorous and appropriate selection of candidates for HTX remains essential to ensure favorable post-transplant outcomes. The underlying etiology leading to HTX may constitute a relevant prognostic factor. Accordingly, we conducted a retrospective single-center observational study including 387 adult patients who underwent HTX at the Heidelberg Heart Center between 1998 and 2020. The study compared post-transplant outcomes between 158 HTX recipients with pre-transplant ICM (40.8%) and 229 HTX recipients with pre-transplant DCM (59.2%). HTX recipients with a pre-transplant etiology of DCM demonstrated a significantly lower 5-year post-transplant mortality compared with HTX recipients with prior ICM (27.5% versus 41.1%, *p* = 0.005). Multivariate analysis incorporating 15 variables identified pre-transplant DCM as an independent predictor of reduced 5-year post-transplant mortality risk (HR: 0.478, 95% CI: 0.235–0.972, *p* = 0.042). Furthermore, subgroup analysis of 62 HTX recipients with pre-transplant familial DCM and 167 recipients with pre-transplant idiopathic DCM showed a significantly lower 5-year post-transplant mortality in HTX recipients with pre-transplant familial DCM (14.5% versus 32.3%, *p* = 0.007). A second multivariate analysis showed that HTX recipients with pre-transplant familial DCM had a significantly lower risk of 5-year post-transplant mortality (HR: 0.380, 95% CI: 0.179–0.807, *p* = 0.012).

In summary, our findings indicate that HTX recipients with a pre-transplant etiology of DCM, particularly familial DCM, exhibit superior 5-year post-transplant survival compared with HTX recipients with pre-transplant ICM. This survival advantage may be attributable to a more favorable cardiovascular risk profile among patients with pre-transplant DCM, although residual confounding cannot be excluded. As the etiology of end-stage HF cannot be fully separated from its associated clinical phenotype, it should not be considered in isolation, but rather as one component of a comprehensive, multiparametric risk assessment during candidate selection. Therefore, rigorous evaluation and optimization of cardiovascular risk factors during the HTX assessment process, supported by targeted preventive and therapeutic interventions, may enhance post-transplant survival outcomes.

## Figures and Tables

**Table 1:** Demographics—stratified by DCM versus ICM before HTX.

Parameter	All Patients (*n* = 387)	DCM Before HTX (*n* = 229)	ICM Before HTX (*n* = 158)	Difference	95% CI	*p*-Value
Recipient data						
Age (years), mean ± SD	52.6 ± 10.4	50.0 ± 11.6	56.4 ± 6.8	6.4	4.6–8.2	<0.001 *
Male sex, *n* (%)	307 (79.3%)	169 (73.8%)	138 (87.3%)	13.5%	5.8–21.2%	0.001 *
BMI (kg/m^2^), mean ± SD	25.5 ± 4.1	25.1 ± 4.1	26.0 ± 4.1	0.9	0.1–1.7	0.038 *
Arterial hypertension, *n* (%)	229 (59.2%)	77 (33.6%)	152 (96.2%)	62.6%	55.8–69.4%	<0.001 *
Dyslipidemia, *n* (%)	274 (70.8%)	119 (52.0%)	155 (98.1%)	46.1%	39.3–52.9%	<0.001 *
Diabetes mellitus, *n* (%)	137 (35.4%)	66 (28.8%)	71 (44.9%)	16.1%	6.4–25.8%	0.001 *
COPD, *n* (%)	108 (27.9%)	47 (20.5%)	61 (38.6%)	18.1%	8.9–27.3%	<0.001 *
History of smoking, *n* (%)	244 (63.0%)	116 (50.7%)	128 (81.0%)	30.3%	21.4–39.2%	<0.001 *
Chronic kidney disease ^, *n* (%)	219 (56.6%)	125 (54.6%)	94 (59.5%)	4.9%	−5.1–14.9%	0.338
eGFR (mL/min/1.73 m^2^), mean ± SD	60.1 ± 24.0	61.2 ± 25.3	58.5 ± 22.1	2.7	−2.1–7.5	0.277
Previous open-heart surgery						
Overall open-heart surgery, *n* (%)	146 (37.7%)	66 (28.8%)	80 (50.6%)	21.8%	12.0–31.6%	<0.001 *
CABG surgery, *n* (%)	63 (16.3%)	0 (0.0%)	63 (39.9%)	39.9%	32.3–47.5%	<0.001 *
Other surgery °, *n* (%)	36 (9.3%)	21 (9.2%)	15 (9.5%)	0.3%	−5.6–6.2%	0.914
VAD surgery, *n* (%)	66 (17.1%)	48 (21.0%)	18 (11.4%)	9.6%	2.4–16.8%	0.014 *
Donor data						
Age (years), mean ± SD	43.4 ± 13.4	41.9 ± 13.7	45.5 ± 12.6	3.6	0.9–6.3	0.009 *
Male sex, *n* (%)	149 (38.5%)	89 (38.9%)	60 (38.0%)	0.9%	−9.0–10.8%	0.860
BMI (kg/m^2^), mean ± SD	25.1 ± 4.4	25.0 ± 4.5	25.3 ± 4.3	0.3	−0.6–1.2	0.510
Transplant sex mismatch						
Mismatch, *n* (%)	188 (48.6%)	106 (46.3%)	82 (51.9%)	5.6%	−4.5–15.7%	0.278
Donor (m) to recipient (f), *n* (%)	15 (3.9%)	13 (5.7%)	2 (1.3%)	4.4%	0.9–7.9%	0.027 *
Donor (f) to recipient (m), *n* (%)	173 (44.7%)	93 (40.6%)	80 (50.6%)	10.0%	−0.1–20.1%	0.051
Perioperative data						
Ischemic time (min), mean ± SD	244.4 ± 60.9	240.3 ± 61.2	250.4 ± 60.0	10.1	−2.2–22.4	0.108
Biatrial anastomosis, *n* (%)	2 (0.5%)	0 (0.0%)	2 (1.3%)	1.3%	−0.5–3.1%	0.088
Bicaval anastomosis, *n* (%)	168 (43.4%)	104 (45.4%)	64 (40.5%)	4.9%	−5.1–14.9%	0.338
Total orthotopic anastomosis, *n* (%)	217 (56.1%)	125 (54.6%)	92 (58.2%)	3.6%	−6.4–13.6%	0.478
High-urgent listing status, *n* (%)	245 (63.3%)	148 (64.6%)	97 (61.4%)	3.2%	−6.6–13.0%	0.516

BMI = body mass index; CABG = coronary artery bypass graft; CI = confidence interval; COPD = chronic obstructive pulmonary disease; DCM = dilated cardiomyopathy; eGFR = estimated glomerular filtration rate; f = female; HTX = heart transplantation; ICM = ischemic cardiomyopathy; m = male; *n* = number; SD = standard deviation; VAD = ventricular assist device; ^ = eGFR < 60 mL/min/1.73 m^2^; ° = congenital, valvular, or ventricular surgery; * = statistically significant (*p* < 0.050).

**Table 2:** Demographics—stratified by familial versus idiopathic DCM before HTX.

Parameter	DCM Before HTX (*n* = 229)	Familial DCM Before HTX (*n* = 62)	Idiopathic DCM Before HTX (*n* = 167)	Difference	95% CI	*p*-Value
Recipient data						
Age (years), mean ± SD	50.0 ± 11.6	46.1 ± 11.1	51.5 ± 11.4	5.4	2.1–8.7	0.001 *
Male sex, *n* (%)	169 (73.8%)	42 (67.7%)	127 (76.0%)	8.3%	−5.0–21.6%	0.204
BMI (kg/m^2^), mean ± SD	25.1 ± 4.1	24.7 ± 4.2	25.3 ± 4.0	0.6	−0.6–1.8	0.334
Arterial hypertension, *n* (%)	77 (33.6%)	17 (27.4%)	60 (35.9%)	8.5%	−4.8–21.8%	0.226
Dyslipidemia, *n* (%)	119 (52.0%)	30 (48.4%)	89 (53.3%)	4.9%	−9.7–19.5%	0.509
Diabetes mellitus, *n* (%)	66 (28.8%)	12 (19.4%)	54 (32.3%)	12.9%	−0.1–25.9%	0.054
COPD, *n* (%)	47 (20.5%)	8 (12.9%)	39 (23.4%)	10.5%	−0.3–21.3%	0.082
History of smoking, *n* (%)	116 (50.7%)	33 (53.2%)	83 (49.7%)	3.5%	−11.1–18.1%	0.635
Chronic kidney disease ^, *n* (%)	125 (54.6%)	28 (45.2%)	97 (58.1%)	12.9%	−1.6–27.4%	0.081
eGFR (ml/min/1.73 m^2^), mean ± SD	61.2 ± 25.3	64.0 ± 27.9	60.1 ± 24.2	3.9	−3.9–11.7	0.331
Previous open-heart surgery						
Overall open-heart surgery, *n* (%)	66 (28.8%)	19 (30.6%)	47 (28.1%)	2.5%	−10.8–15.8%	0.710
CABG surgery, *n* (%)	0 (0.0%)	0 (0.0%)	0 (0.0%)	0.0%	n. a.	n. a.
Other surgery °, *n* (%)	21 (9.2%)	4 (6.5%)	17 (10.2%)	3.7%	−4.0–11.4%	0.385
VAD surgery, *n* (%)	48 (21.0%)	15 (24.2%)	33 (19.8%)	4.4%	−7.9–16.7%	0.464
Donor data						
Age (years), mean ± SD	41.9 ± 13.7	45.8 ± 10.7	40.5 ± 14.4	5.3	1.9–8.7	0.003 *
Male sex, *n* (%)	89 (38.9%)	13 (21.0%)	76 (45.5%)	24.5%	11.9–37.1%	0.001 *
BMI (kg/m^2^), mean ± SD	25.0 ± 4.5	25.2 ± 4.9	24.9 ± 4.3	0.3	−1.1–1.7	0.663
Transplant sex mismatch						
Mismatch, *n* (%)	106 (46.3%)	33 (53.2%)	73 (43.7%)	9.5%	−5.0–24.0%	0.200
Donor (m) to recipient (f), *n* (%)	13 (5.7%)	2 (3.2%)	11 (6.6%)	3.4%	−2.4–9.2%	0.329
Donor (f) to recipient (m), *n* (%)	93 (40.6%)	31 (50.0%)	62 (37.1%)	12.9%	−1.5–27.3%	0.078
Perioperative data						
Ischemic time (min), mean ± SD	240.3 ± 61.2	255.9 ± 60.4	234.6 ± 60.7	21.3	3.7–38.9	0.020 *
Biatrial anastomosis, *n* (%)	0 (0.0%)	0 (0.0%)	0 (0.0%)	0.0%	n. a.	n. a.
Bicaval anastomosis, *n* (%)	104 (45.4%)	34 (54.8%)	70 (41.9%)	12.9%	−1.6–27.4%	0.081
Total orthotopic anastomosis, *n* (%)	125 (54.6%)	28 (45.2%)	97 (58.1%)	12.9%	−1.6–27.4%	0.081
High-urgent listing status, *n* (%)	148 (64.6%)	46 (74.2%)	102 (61.1%)	13.1%	−0.1–26.3%	0.065

BMI = body mass index; CABG = coronary artery bypass graft; CI = confidence interval; COPD = chronic obstructive pulmonary disease; DCM = dilated cardiomyopathy; eGFR = estimated glomerular filtration rate; f = female; HTX = heart transplantation; m = male; *n* = number; n. a. = not applicable; SD = standard deviation; VAD = ventricular assist device; ^ = eGFR < 60 mL/min/1.73 m^2^; ° = congenital, valvular, or ventricular surgery; * = statistically significant (*p* < 0.050).

**Table 3:** Initial medications after HTX—stratified by DCM versus ICM before HTX.

Parameter	All Patients (*n* = 387)	DCM Before HTX (*n* = 229)	ICM Before HTX (*n* = 158)	Difference	95% CI	*p*-Value
Immunosuppressive therapy						
Cyclosporine A, *n* (%)	149 (38.5%)	89 (38.9%)	60 (38.0%)	0.9%	−9.0–10.8%	0.860
Tacrolimus, *n* (%)	238 (61.5%)	140 (61.1%)	98 (62.0%)	0.9%	−9.0–10.8%	0.860
Azathioprine, *n* (%)	82 (21.2%)	49 (21.4%)	33 (20.9%)	0.5%	−7.8–8.8%	0.904
Mycophenolic acid, *n* (%)	305 (78.8%)	180 (78.6%)	125 (79.1%)	0.5%	−7.8–8.8%	0.904
Steroids, *n* (%)	387 (100.0%)	229 (100.0%)	158 (100.0%)	0.0%	n. a.	n. a.
Concomitant medications						
ASA, *n* (%)	63 (16.3%)	37 (16.2%)	26 (16.5%)	0.3%	−7.2–7.8%	0.938
Beta-blocker, *n* (%)	76 (19.6%)	37 (16.2%)	39 (24.7%)	8.5%	0.2–16.8%	0.038 *
Ivabradine, *n* (%)	51 (13.2%)	35 (15.3%)	16 (10.1%)	5.2%	−1.4–11.8%	0.140
Calcium channel blocker, *n* (%)	124 (32.0%)	67 (29.3%)	57 (36.1%)	6.8%	−2.7–16.3%	0.158
ACE inhibitor/ARB, *n* (%)	187 (48.3%)	119 (52.0%)	68 (43.0%)	9.0%	−1.1–19.1%	0.084
Diuretic, *n* (%)	387 (100.0%)	229 (100.0%)	158 (100.0%)	0.0%	n. a.	n. a.
Statin, *n* (%)	218 (56.3%)	120 (52.4%)	98 (62.0%)	9.6%	−0.4–19.6%	0.061
Gastric protection drug ^†^, *n* (%)	387 (100.0%)	229 (100.0%)	158 (100.0%)	0.0%	n. a.	n. a.

ACE inhibitor = angiotensin-converting enzyme inhibitor; ARB = angiotensin II receptor blocker; ASA = acetylsalicylic acid; CI = confidence interval; DCM = dilated cardiomyopathy; HTX = heart transplantation; ICM = ischemic cardiomyopathy; *n* = number; n. a. = not applicable; ^†^ = gastric protection drug defined as proton pump inhibitor (PPI) or histamine receptor (H_2_) blocker; * = statistically significant (*p* < 0.050).

**Table 4:** Initial medications after HTX—stratified by familial versus idiopathic DCM before HTX.

Parameter	DCM Before HTX (*n* = 229)	Familial DCM Before HTX (*n* = 62)	Idiopathic DCM Before HTX (*n* = 167)	Difference	95% CI	*p*-Value
Immunosuppressive therapy						
Cyclosporine A, *n* (%)	89 (38.9%)	18 (29.0%)	71 (42.5%)	13.5%	−0.1–27.1%	0.063
Tacrolimus, *n* (%)	140 (61.1%)	44 (71.0%)	96 (57.5%)	13.5%	−0.1–27.1%	0.063
Azathioprine, *n* (%)	49 (21.4%)	5 (8.1%)	44 (26.3%)	18.2%	8.7–27.7%	0.003 *
Mycophenolic acid, *n* (%)	180 (78.6%)	57 (91.9%)	123 (73.7%)	18.2%	8.7–27.7%	0.003 *
Steroids, *n* (%)	229 (100.0%)	62 (100.0%)	167 (100.0%)	0.0%	n. a.	n. a.
Concomitant medications						
ASA, *n* (%)	37 (16.2%)	12 (19.4%)	25 (15.0%)	4.4%	−6.8–15.6%	0.423
Beta-blocker, *n* (%)	37 (16.2%)	17 (27.4%)	20 (12.0%)	15.4%	3.2–27.6%	0.005 *
Ivabradine, *n* (%)	35 (15.3%)	11 (17.7%)	24 (14.4%)	3.3%	−7.6–14.2%	0.529
Calcium channel blocker, *n* (%)	67 (29.3%)	10 (16.1%)	57 (34.1%)	18.0%	6.4–29.6%	0.008 *
ACE inhibitor/ARB, *n* (%)	119 (52.0%)	34 (54.8%)	85 (50.9%)	3.9%	−10.6–18.4%	0.596
Diuretic, *n* (%)	229 (100.0%)	62 (100.0%)	167 (100.0%)	0.0%	n. a.	n. a.
Statin, *n* (%)	120 (52.4%)	36 (58.1%)	84 (50.3%)	7.8%	−6.6–22.2%	0.296
Gastric protection drug ^†^, *n* (%)	229 (100.0%)	62 (100.0%)	167 (100.0%)	0.0%	n. a.	n. a.

ACE inhibitor = angiotensin-converting enzyme inhibitor; ARB = angiotensin II receptor blocker; ASA = acetylsalicylic acid; CI = confidence interval; DCM = dilated cardiomyopathy; HTX = heart transplantation; *n* = number; n. a. = not applicable; ^†^ = gastric protection drug defined as proton pump inhibitor (PPI) or histamine receptor (H_2_) blocker; * = statistically significant (*p* < 0.050).

**Table 5:** 5-year cause-specific mortality after HTX—stratified by DCM versus ICM before HTX.

Parameter	All Patients (*n* = 387)	DCM Before HTX (*n* = 229)	ICM Before HTX (*n* = 158)	Difference	95% CI	*p*-Value
Graft failure, *n* (%)	33 (8.5%)	17 (7.4%)	16 (10.1%)	2.7%	−3.1–8.5%	0.349
Acute rejection, *n* (%)	4 (1.1%)	3 (1.3%)	1 (0.6%)	0.7%	−1.2–2.6%	0.517
Infection/Sepsis, *n* (%)	67 (17.3%)	32 (14.0%)	35 (22.2%)	8.2%	0.3–16.1%	0.037 *
Malignancy, *n* (%)	12 (3.1%)	6 (2.6%)	6 (3.8%)	1.2%	−2.4–4.8%	0.511
Thromboembolic event/bleeding, *n* (%)	12 (3.1%)	5 (2.2%)	7 (4.4%)	2.2%	−1.5–5.9%	0.210
All causes, *n* (%)	128 (33.1%)	63 (27.5%)	65 (41.1%)	13.6%	4.0–23.2%	0.005 *

CI = confidence interval; DCM = dilated cardiomyopathy; HTX = heart transplantation; ICM = ischemic cardiomyopathy; *n* = number; * = statistically significant (*p* < 0.050).

**Table 6:** 5-year cause-specific mortality after HTX—stratified by familial versus idiopathic DCM before HTX.

Parameter	DCM Before HTX (*n* = 229)	Familial DCM Before HTX (*n* = 62)	Idiopathic DCM Before HTX (*n* = 167)	Difference	95% CI	*p*-Value
Graft failure, *n* (%)	17 (7.4%)	2 (3.2%)	15 (9.0%)	5.8%	−0.4–12.0%	0.140
Acute rejection, *n* (%)	3 (1.3%)	0 (0.0%)	3 (1.8%)	1.8%	−0.2–3.8%	0.288
Infection/Sepsis, *n* (%)	32 (14.0%)	6 (9.7%)	26 (15.5%)	5.8%	−3.4–15.0%	0.253
Malignancy, *n* (%)	6 (2.6%)	0 (0.0%)	6 (3.6%)	3.6%	−0.2–7.4%	0.130
Thromboembolic event/bleeding, *n* (%)	5 (2.2%)	1 (1.6%)	4 (2.4%)	0.8%	−3.1–4.7%	0.719
All causes, *n* (%)	63 (27.5%)	9 (14.5%)	54 (32.3%)	17.8%	6.5–29.1%	0.007 *

CI = confidence interval; DCM = dilated cardiomyopathy; HTX = heart transplantation; *n* = number; * = statistically significant (*p* < 0.050).

**Table 7:** Multivariate analysis for 5-year mortality after HTX—focus on DCM before HTX.

Parameter	Hazard Ratio	95% CI	*p*-Value
Recipient DCM before HTX (in total)	0.478	0.235–0.972	0.042 *
Recipient age (years)	1.031	1.007–1.055	0.011 *
Recipient male sex (in total)	1.009	0.594–1.713	0.974
Recipient BMI (kg/m^2^)	0.999	0.954–1.047	0.991
Recipient arterial hypertension (in total)	0.754	0.418–1.359	0.347
Recipient dyslipidemia (in total)	0.824	0.470–1.445	0.499
Recipient diabetes mellitus (in total)	1.200	0.823–1.749	0.343
Recipient COPD (in total)	4.202	2.736–6.452	<0.001 *
Recipient history of smoking (in total)	0.766	0.463–1.265	0.297
Recipient overall open-heart surgery (in total)	1.998	1.098–3.636	0.023 *
Recipient CABG surgery (in total)	0.505	0.247–1.035	0.062
Recipient VAD surgery (in total)	0.877	0.464–1.655	0.685
Donor age (years)	1.009	0.995–1.024	0.212
Donor (m) to recipient (f) transplant mismatch (in total)	2.419	0.986–5.936	0.054
Recipient beta-blocker therapy after HTX (in total)	0.821	0.519–1.299	0.400

BMI = body mass index; CABG = coronary artery bypass graft; CI = confidence interval; COPD = chronic obstructive pulmonary disease; DCM = dilated cardiomyopathy; f = female; HTX = heart transplantation; m = male; VAD = ventricular assist device; * = statistically significant (*p* < 0.050).

**Table 8:** Multivariate analysis for 5-year mortality after HTX—focus on familial DCM before HTX.

Parameter	Hazard Ratio	95% CI	*p*-Value
Recipient familial DCM before HTX (in total)	0.380	0.179–0.807	0.012 *
Recipient age (years)	1.021	0.996–1.047	0.102
Donor age (years)	1.004	0.984–1.024	0.694
Donor male sex (in total)	0.552	0.317–0.961	0.036 *
Ischemic time (min)	1.006	1.001–1.011	0.017 *
Recipient azathioprine therapy after HTX (in total)	2.541	1.192–5.418	0.016 *
Recipient beta-blocker therapy after HTX (in total)	1.392	0.711–2.724	0.334
Recipient calcium channel blocker therapy after HTX (in total)	0.911	0.524–1.582	0.740

CI = confidence interval; DCM = dilated cardiomyopathy; HTX = heart transplantation; * = statistically significant (*p* < 0.050).

**Table 9:** Cardiac catheterization after HTX—stratified by DCM versus ICM before HTX.

Parameter	All Patients (*n* = 387)	DCM Before HTX (*n* = 229)	ICM Before HTX (*n* = 158)	Difference	95% CI	*p*-Value
CAD (stenosis ≥ 50%), *n* (%)						
Within 1 year after HTX	17 (4.4%)	12 (5.2%)	5 (3.2%)	2.0%	−2.0–6.0%	0.327
Within 2 years after HTX	48 (12.4%)	28 (12.2%)	20 (12.7%)	0.5%	−6.2–7.2%	0.899
Within 5 years after HTX	75 (19.4%)	44 (19.2%)	31 (19.6%)	0.4%	−7.6–8.4%	0.921
*p*-value: within 1 year versus within 2 years	<0.001 *	0.008 *	0.002 *			
*p*-value: within 1 year versus within 5 years	<0.001 *	<0.001 *	<0.001 *			
Coronary stenting, *n* (%)						
Within 1 year after HTX	4 (1.0%)	3 (1.3%)	1 (0.6%)	0.7%	−1.2–2.6%	0.517
Within 2 years after HTX	18 (4.7%)	14 (6.1%)	4 (2.5%)	3.6%	−0.3–7.5%	0.100
Within 5 years after HTX	31 (8.0%)	19 (8.3%)	12 (7.6%)	0.7%	−4.8–6.2%	0.803
*p*-value: within 1 year versus within 2 years	0.002 *	0.007 *	0.176			
*p*-value: within 1 year versus within 5 years	<0.001 *	<0.001 *	0.002 *			

CAD = coronary artery disease; CI = confidence interval; DCM = dilated cardiomyopathy; HTX = heart transplantation; ICM = ischemic cardiomyopathy; *n* = number; * = statistically significant (*p* < 0.050).

**Table 10:** Cardiac catheterization after HTX—stratified by familial versus idiopathic DCM before HTX.

Parameter	DCM Before HTX (*n* = 229)	Familial DCM Before HTX (*n* = 62)	Idiopathic DCM Before HTX (*n* = 167)	Difference	95% CI	*p*-Value
CAD (stenosis ≥ 50%), *n* (%)						
Within 1 year after HTX	12 (5.2%)	6 (9.7%)	6 (3.6%)	6.1%	−1.8–14.0%	0.066
Within 2 years after HTX	28 (12.2%)	8 (12.9%)	20 (12.0%)	0.9%	−8.8–10.6%	0.849
Within 5 years after HTX	44 (19.2%)	12 (19.4%)	32 (19.2%)	0.2%	−11.3–11.7%	0.974
*p*-value: within 1 year versus within 2 years	0.008 *	0.570	0.004 *			
*p*-value: within 1 year versus within 5 years	<0.001 *	0.126	<0.001 *			
Coronary stenting, *n* (%)						
Within 1 year after HTX	3 (1.3%)	2 (3.2%)	1 (0.6%)	2.6%	−1.9–7.1%	0.120
Within 2 years after HTX	14 (6.1%)	5 (8.1%)	9 (5.4%)	2.7%	−4.9–10.3%	0.453
Within 5 years after HTX	19 (8.3%)	7 (11.3%)	12 (7.2%)	4.1%	−4.7–12.9%	0.317
*p*-value: within 1 year versus within 2 years	0.007 *	0.243	0.010 *			
*p*-value: within 1 year versus within 5 years	<0.001 *	0.084	0.002 *			

CAD = coronary artery disease; CI = confidence interval; DCM = dilated cardiomyopathy; HTX = heart transplantation; *n* = number; * = statistically significant (*p* < 0.050).

## Data Availability

The original contributions presented in this study are included in this article; further inquiries can be directed to the corresponding author.
